# Facile Access to Solifenacin Impurity K: One-Step Synthesis and an HPLC-MS Method for Its Determination

**DOI:** 10.3390/molecules29133011

**Published:** 2024-06-25

**Authors:** Raúl Xifra, Andrés E. Lukach, Andreea L. Turcu

**Affiliations:** 1 Research and Development Center for Organic Chemistry, (CIDQO, S.L.), Polígono Industrial “Can Verdalet”, Calle D, Nave 91, 08490 Tordera, Spain; rxifra@ewatts-tech.com (R.X.); alukach@ewatts-tech.com (A.E.L.); 2 Laboratory of Medicinal Chemistry (Associated Unit to CSIC), Faculty of Pharmacy and Food Sciences, University of Barcelona, Avinguda Joan XXIII, 27-31, 08028 Barcelona, Spain; 3 Institute of Biomedicine of the University of Barcelona (IBUB), University of Barcelona, 08028 Barcelona, Spain

**Keywords:** solifenacin succinate, impurity K, HPLC-MS analytical method

## Abstract

Solifenacin (SFC) is a potent muscarinic antagonist that effectively reduces bladder muscle contraction, thereby alleviating symptoms such as frequency of micturition and urgency. Oxidation of SFC leads to the formation of impurities like Impurity K. Effective analysis and control of this impurity is crucial for ensuring compliance with regulatory standards and safeguarding patient health. To address these challenges, we propose a novel one-step synthesis of Impurity K from SFC. Impurity K was synthesized using cerium(IV) ammonium nitrate (CAN) in water/acetonitrile as the solvent. Additionally, we describe a new HPLC-MS method for the detection of Impurity K in solifenacin succinate tablets.

## 1. Introduction

Solifenacin (SFC), developed by Yamanouchi Pharmaceuticals (now part of Astellas Pharma), is a competitive muscarinic antagonist with a higher affinity for the M3 subtype. Its mechanism of action involves the reduction of smooth muscle contraction in the bladder wall, resulting in a decrease in the frequency of micturition, urgency, and episodes of incontinence [1]. Marketed as solifenacin succinate (SLN; YM905; Vesicare^®^), it was initially approved in 2004 for the treatment of overactive bladder in adults. More recently, the FDA has granted approval for the treatment of neurogenic detrusor overactivity, a form of bladder dysfunction related to neurological impairment, in children aged two years and older [2].

**Scheme 1:**
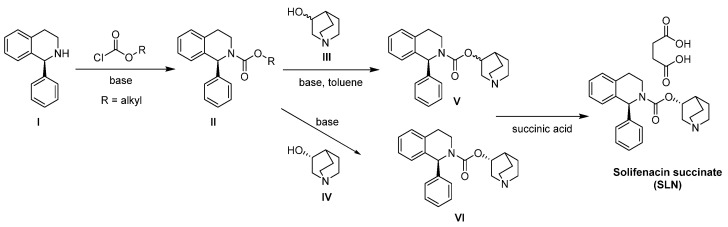
Two routes of synthesis of solifenacin succinate (SLN).

SFC, also known as (1*S*,3*R*)-1-azabicyclo [2.2.2]oct-3-yl-3,4-dihydro-1-phenyl-2(1*H*)-isoquinolinecarboxylate is a chiral compound that could be synthesized through a multi-step process starting from (1*S*)-1-phenyl-1,2,3,4-tetrahydroisoquinoline (**I**) (Scheme 1). The condensation of **I** with alkyl chloroformate yields carbamate **II**, which reacts with racemic 3-quinuclidinol to generate a diastereomeric mixture of SFC (**V**). Treating this mixture with succinic acid in a solvent blend results in optically pure SLN [3]. Alternatively, intermediate **II** can react with (*R*)-3-quinuclidinol to produce optically pure SFC (**VI**), which, upon treatment with succinic acid, yields enantiopure SLN [4].

**Figure 1:**
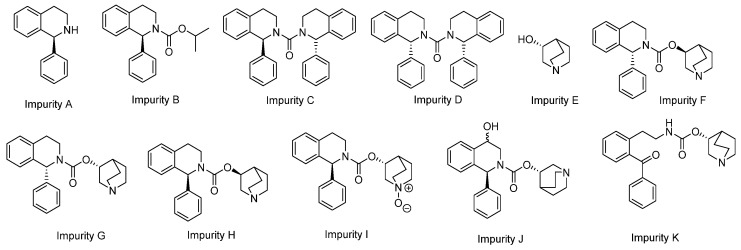
Structures of SFC impurities resulting from its synthesis process or degradation [5,11,12].

Although these synthetic routes are efficient, they may result in process-related impurities. In fact, most impurities described in the literature are formed during the synthesis process (e.g., impurities A–H, Figure 1). Others may arise from SFC degradation following processes such as oxidation, as seen in Impurities I–K (Figure 1) [5,6,7,8,9,10,11,12,13].

**Scheme 2:**
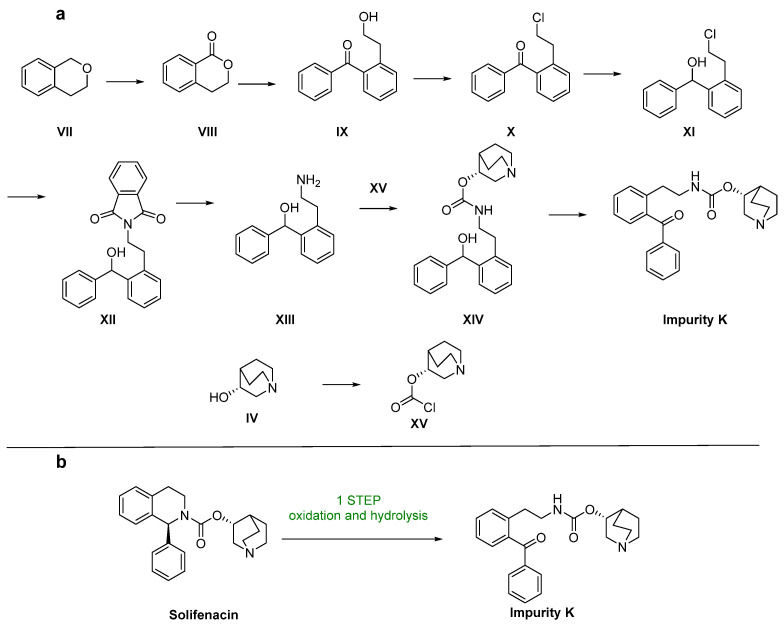
(**a**) Previously reported route for the nine-step synthesis of Impurity K. (**b**) Present methodology for the synthesis of Impurity K in one step.

Regarding the impurities resulting from SFC degradation, the most commonly observed and identified impurity is *N*-oxide SFC (Impurity I), while studies on other impurities are still limited. Impurity K, resulting from the oxidation of SFC at the double benzylic position, was detected in SLN tablets by Do Hwan Kim & Co., and efforts have been made to minimize its presence by modifying the tablet fabrication process [14]. However, despite the presence of this impurity in the SLN tablets, only a complex synthetic route for its synthesis is reported in the literature [12]. This route involves a nine-step synthesis starting from 3,4-dihydro-1*H*-benzo[*c*]pyran, with an overall yield of around 2% (Scheme 2a). Furthermore, there is currently no HPLC-MS method for the determination of Impurity K.

The presence of the impurities, even in small quantities, can reduce the effectiveness of dosage forms and pose risks to patient safety. Therefore, accurate analysis and identification of these impurities are compulsory to ensure proper control, with limits not exceeding that recommended by regulatory authorities [15,16]. Consequently, access to standard impurities is critical for their accurate identification.

For this reason, we present a one-step reaction for synthesizing Impurity K from SFC (Scheme 2b) through a novel, faster, and simplified procedure. Additionally, we propose a new HPLC method suitable for HPLC-MS for the easy detection and identification of Impurity K in solifenacin succinate tablets. This method enables differentiation from other products of degradation resulting from oxidation, such as Impurity I.

## 2. Results and Discussion

### 2.1. Synthesis of Impurity K

It is known that the oxidation of several tetrahydroisoquinoline alkaloids with CAN in methanol/acetonitrile resulted in the stereoselective introduction of a methoxy group at a benzylic position [17,18]. Considering these precedents, we hypothesized that the oxidation of SFC may lead to the formation of Impurity K.

**Scheme 3:**
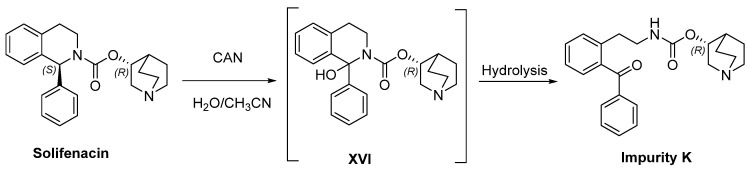
Oxidation of SFC with CAN in a mixture of acetonitrile and water followed by hydrolysis.

Indeed, the treatment of SFC with cerium ammonium nitrate (CAN) in water/acetonitrile led to Impurity K, very likely through the intermediacy of alcohol **XVI** (Scheme 3). This intermediate should undergo hydrolysis to the corresponding ketone. HPLC analysis of the crude revealed the presence of solifenacin *N*-oxide (Impurity I), the desired Impurity K, and other minor byproducts. Following a work-up and column chromatography purification, Impurity K was obtained with an 18% yield and a purity > 95.0%. Therefore, we have not only improved the previously reported synthetic pathway for attaining Impurity K, reducing the number of steps involved, but we have also significantly enhanced the yield, from the around 2% reported in the literature to the currently obtained 18% yield.

### 2.2. HPLC-MS Method for the Analysis of Impurity K

**Figure 2:**
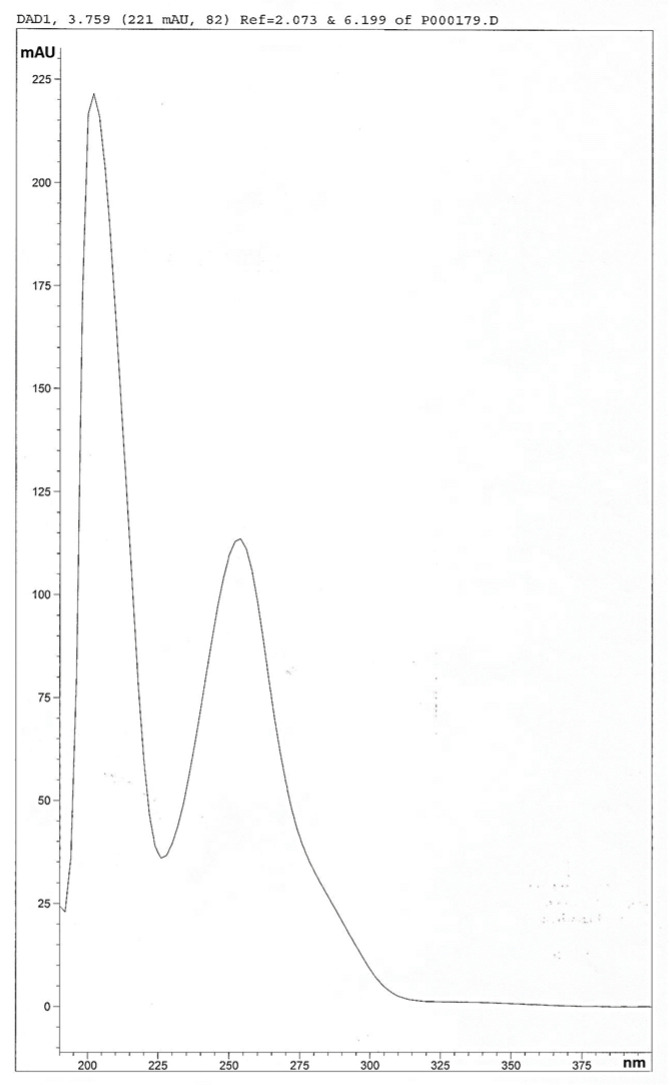
The UV spectra of Impurity K.

Importantly, once Impurity K was synthesized, an HPLC-MS method for its identification and differentiation from Impurity I, which has the same molecular weight, was designed and developed. Standard of Impurity I has been synthesized by us following the procedure already reported [19]. Thus, SFC and Impurities I and K were distinguished firstly by comparison of the retention times of the three compounds separately by using an XBridge C18 (Waters) column 250 × 4.6 mm (particle size: 5 µM) as the stationary phase and a buffer of ammonium acetate and acetonitrile at pH = 7 as the mobile phase, with a UV detector set at 220 nm (Table 1). Secondly, the UV spectra of the three compounds were compared. While solifenacin and Impurity I exhibited a broad, strong peak at 210 nm, Impurity K displayed an additional prominent band at 250 nm. This secondary band suggests increased conjugation within the molecule, a characteristic of the benzophenone unit of Impurity K (Figure 2).

Furthermore, we prepared and analyzed a sample of SLN tablets by HPLC-MS, observing that while Impurity I was present at about 1.95%, Impurity K was at approximately 0.88% (see Section 3 and Supplementary Materials for further details). This finding confirms that our method is capable of detecting Impurity K in SLN tablets and differentiating it from impurity I.

### 2.3. Characterization of Impurity K

Impurity K was fully characterized by ^1^H, ^13^C, DEPT, HSQC, COSY NMR experiments, infrared, UV spectrum, GC/MS (EI) spectrum, and melting point (see Section 3 and Supplementary Materials for further details).

## 3. Materials and Methods

### 3.1. Chemical Synthesis

#### 3.1.1. General Methods

Commercially available reagents and solvents were used without further purification unless stated otherwise. Melting points were determined in open capillary tubes. NMR spectra were recorded in CDCl_3_ in a Varian Mercury (400 MHz, ^1^H NMR; 100.6 MHz, ^13^C NMR). Chemical shifts (δ) are reported in ppm related to internal tetramethylsilane (TMS). Assignments given for the NMR spectra are based on chemical shifts, coupling constants, COSY, HSQC, and DEPT. The HPLC studies were carried out in an Agilent 1200 (with a quaternary pump, on-line degasser, and DAD detector) using a Waters Xbridge C18 column. For the GC/MS (EI), the sample was introduced directly in a Hewlett–Packard 5988A. The electron impact (70 eV) technique was used. Only significant ions are given, i.e., those with a higher relative ratio, except for the ions with higher *m*/*z* values. The IR spectrum was recorded in a Perkin Elmer Spectrum RX I. Absorption values in the IR spectrum (KBr) are given as wave numbers (cm^−1^). Only the more intense bands are given. Column chromatographies were performed on silica gel 60 Å (35–70 mesh). For the thin layer chromatography (TLC), aluminum-backed sheets with silica gel 60 F_254_ were used, and spots were visualized with UV light and/or a 1% aqueous solution of KMnO_4_.

#### 3.1.2. Synthesis and Characterization Data of Impurity K

To a solution of solifenacin (6.50 g, 17.9 mmol) in acetonitrile (19.5 mL) and water (32.5 mL), cerium(IV) ammonium nitrate (CAN) (21.65 g, 39.50 mmol) was added, and the reaction mixture was heated to 70–75 °C. After 2 h, the pale-yellow solution still contained solifenacin (HPLC control). A second portion of using cerium(IV) ammonium nitrate (29.52 g, 53.85 mmol) was added, and the mixture was heated at 70–75 °C for 1 h in order to consume all the solifenacin. The mixture was cooled to room temperature, and dichloromethane (65 mL) was added. The aqueous layer was discarded. The organic layer was washed with water (65 mL), aqueous NaHCO_3_ solution (3 × 65 mL), and finally, another time with water (65 mL). The organic layer was dried over anhydrous sodium sulfate, filtered, and concentrated in vacuo to give a foamy residue (4.30 g) that was subjected to flash chromatography on silica gel (160 g of silica gel, mixtures of dichloromethane/methanol) to afford the desired product as a foamy solid (1.50 g, 22% yield). HPLC analysis showed mainly the expected product (>92.0% HPLC) and other unknown impurities. This product was subject to a second flash chromatography on silica gel (60 g of silica gel, mixtures of dichloromethane/methanol) to afford the desired product as a pale-yellow oil (1.2 g, 18% yield) that was solidified by trituration with diethyl ether. HPLC analysis of the solid showed to be the expected product with purity higher than 95.0%, mp. 133–136 °C. IR (KBr): 2963 and 2927 (C-H st), 1708 (C=O st), 1661 (C=O st), 1555, 1451, 1315, 1267, 1134, 1079, 1025, 980, 929, 765, 751, 715, 688, 641 cm^−1^. ^1^H NMR (400 MHz, CDCl_3_): 1.32 (m, 1 H, 5′-H_a_), 1.51 (m, 1 H, 8′-H*_syn_*), 1.63 (m, 1 H, 8′-H*_anti_*), 1.77 (m, 1 H, 5′-H_b_), 1.94 (m, 1 H, 4′-H), 2.61 (dt, *J* = 15 Hz, *J’* = 2 Hz, 2′-H*_endo_*), 2.67–2.85 (complex signal, 4 H, 6-H’_2_ and 7-H’_2_), 2.88 (t, *J* = 7 Hz, 2 H, 2-H_2_), 3.16 (dddd, *J* = 15 Hz, *J’* = 8.4 Hz, *J*” = 2.0 Hz, 1 H, 2-H’*_exo_*), 3.46 (dt, *J* = 7 Hz, *J*’ = 5.6 Hz, 2 H, 1-H_2_), 4.64 (m, 1 H, 3-H’), 5.40 (t, *J* = 5.6 Hz, 1 H, N-H), 7.30 (dd, *J* = 7.2 Hz, *J*’ = 1.2 Hz, 1 H, Ar-H), 7.33 (dd, *J* = 7.2 Hz, *J*’ = 1.8 Hz, 1 H, Ar-H), 7.39 (bd, *J* = 7.6 Hz, 1 H, Ar-H), 7.44–7.49 (complex signal, 3 H, 2 H*_meta_* and Ar-H), 7.60 (tt, *J* = 7.2, *J*’ = 1.2 Hz, 1 H, H*_para_*), 7.80 (dd, *J* = 8.4, *J*’ = 1.2 Hz, 2 H, 2 H*_ortho_*).^13^C NMR (100.6 MHz, CDCl_3_): 19.5 (CH_2_, C5′), 24.6 (CH_2_, C8′), 25.4 (CH, C4′), 33.0 (CH_2_, C2), 42.5 (CH_2_, C1), 46.5 (CH_2_) and 47.4 (CH_2_) (C6′ and C7′), 55.6 (CH_2_, C2′), 71.3 (CH, C3′), 125.7 (CH), 129.1 (CH), 130.7 (CH) and 130.8 (CH) (Ar-3″,4″,5″,6″), 128.4 (CH, C*_meta_*), 130.3 (CH, C*_ortho_*), 133.3 (CH, C*_para_*), 137.6 (C, C-1″), 138.5 (2 C, C-2″ and C*_ipso_*), 156.4 [C, NHC(O)O], 198.4 [C, ArC(O)Ar]. Calcd. for C_23_H_26_N_2_O_3_: C, 72.99; H, 6.92; N, 7.40. Found: C, 72.99; H, 7.06; N, 7.37. GC/MS (EI) m/e (relative intensity): 378 (M^·+^, 17), 209 (29), 208 (43), 207 (16), 195 (27), 194 (29), 165 (22), 127 (22), 126 (100), 110 (51), 109 (97), 105 (17), 98 (15), 81 (22), 77 (20), 55 (10). Ultraviolet spectra: Maxima at 212 and 254 nm.

### 3.2. HPLC Equipment and Methods

#### 3.2.1. HPLC Equipment

An Agilent 1200 Series HPLC system with a quaternary pump coupled to a UV detector set at 220 nm was used. The ESI-MS analyses were recorded on an esquire 6000 ESI ion Trap LC/MS (Bruker Daltonics, Billerica, MA, USA) equipped with an electrospray ion source. The instrument was operated in the positive ESI(+) ion mode. Nitrogen was employed as both a drying and nebulizing gas.

#### 3.2.2. HPLC Method for the Identification of Impurity K

A sample solution of solifenacin succinate (5 mg) was used in this experiment. To a 25 mL volumetric flask were added 10 tablets of SLN. Subsequently, 12.5 mL of NaOH solution with a pH of 11 was added, followed by a 10-min sonication period. Then, 5 mL of ethanol and 1 mL of DMF were introduced into the flask, followed by another 10-min sonication cycle. Acetonitrile was used to make up the volume, followed by an additional 10 min of sonication. The suspension was filtered using a 0.45 µm Nylon filter. The initial 2 mL of the filtered solution were discarded.

Chromatographic conditions were as follows: The employed column was an XBridge C-18 (Waters), 250 mm × 4.5 mm (5 μm), with a constant flow of 1.0 mL min^−1^ and a controlled temperature of 30 °C. The injection volume was 5 μL, and the detection wavelength was set at λ = 220 nm. The mobile phase consisted of pH7 ammonium acetate buffer and acetonitrile, 50:50. pH7 ammonium acetate buffer was prepared from 1.23 g ammonium acetate, 1 L of water, and adjusted pH at 7 with ammonia or acetic acid.

## 4. Conclusions

In conclusion, our study presents a highly improved synthetic protocol for Impurity K characterized by a one-step reaction and improved yield, addressing the limitations of the existing route described in the literature. Through this approach, we successfully synthesized and fully characterized Impurity K. Additionally, we developed an HPLC-MS method specifically designed to accurately identify Impurity K in solifenacin succinate tablets. These advancements offer a faster, simplified, and more efficient approach to the synthesis and detection of this impurity.

## Figures and Tables

**Table 1:** Retention times of SFC, Impurity I, and Impurity K.

Compound	Retention Time (min)
SFC	5.2
Impurity I	3.6
Impurity K	3.9

## Data Availability

Data are contained within the article and Supplementary Materials.

## Supplementary Materials

The following supporting information can be downloaded at: https://www.mdpi.com/article/10.3390/molecules29133011/s1. Figure S1: ^1^H, ^13^C, DEPT, HSQC, COSY, IR, and GC/MS (EI) of Impurity K; Figure S2: HPLC/MS-MS of solifenacin succinate; Figure S3: HPLC/MS-MS of Impurity I; Figure S4: HPLC/MS-MS of Impurity K.
