# Supplementary material for: Facile Access to Solifenacin Impurity K: One-Step Synthesis and an HPLC-MS Method for Its Determination
Source: Molecules. 2024 Jun 25;29(13):3011. doi: 10.3390/molecules29133011 (PMC11243011; doi:10.3390/molecules29133011)
Supplement: Supplementary file 1 [file molecules-29-03011-s001.zip › molecules-3057479-supplementary.pdf]

molecules

SUPPLEMENTARY MATERIAL FOR

Facile Access to Solifenacin Impurity K: One-Step Synthesis and an HPLC-MS method for its determination

Raúl Xifra[1] and Andreea L. Turcu[2,3]*

[1] Centro de investigación y desarrollo para la Química Orgánica S.L. (CIDQO, S. L.); Polígono Industrial "Can Verdalet". Calle D; Nave 91, 08490 Tordera, Spain; rxifra@ewatts-tech.com

[2] Laboratori de Química Farmacèutica (Unitat Associada al CSIC), Facultat de Farmàcia i Ciències de l'Alimentació, Universitat de Barcelona, Av. Joan XXIII, 27-31, 08028 Barcelona, Spain; aturcu@ub.edu

[3] Institute of Biomedicine of the University of Barcelona (IBUB), Universitat de Barcelona, Barcelona, Spain.

Table of contents

Figure S1: ^{1}H, ^{13}C, DEPT, HSQC, COSY and IR GC/MS (EI) of Impurity K Page S3

Figure S2: HPLC/MS-MS of Solifenacin succinate tables Page S13

Figure S3: HPLC/MS-MS of Impurity I Page S15

Figure S4: HPLC/MS-MS of Impurity K Page S17

^{1}H, ^{13}C, DEPT, HSQC, COSY, IR, GC/MS (EI) and UV spectrum of Impurity K

^{1}H (400 MHz, $CDCl_3$)

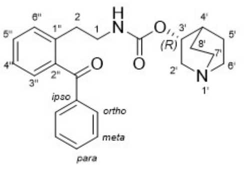

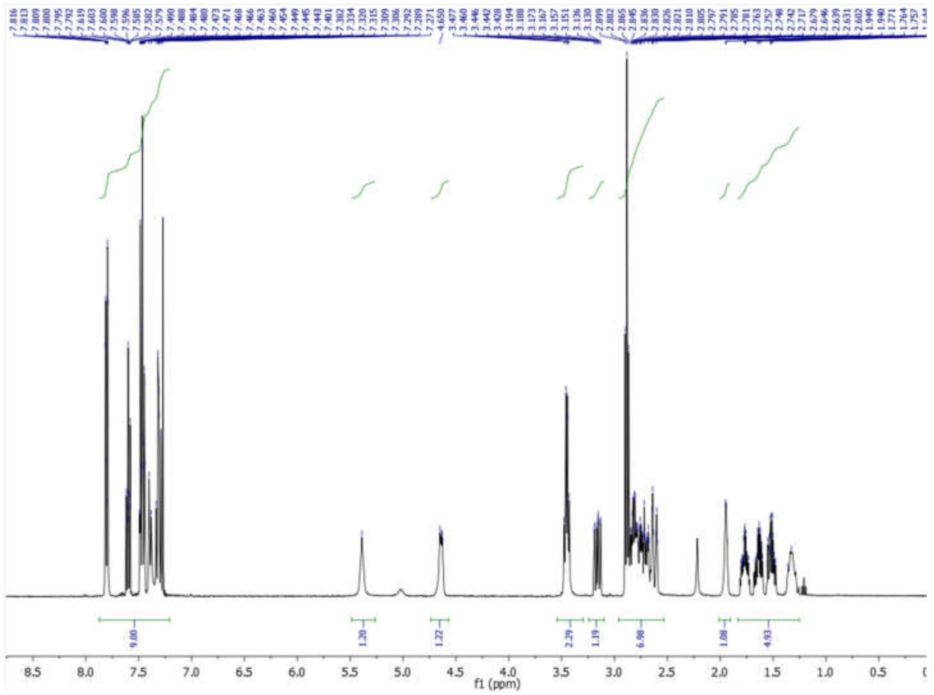

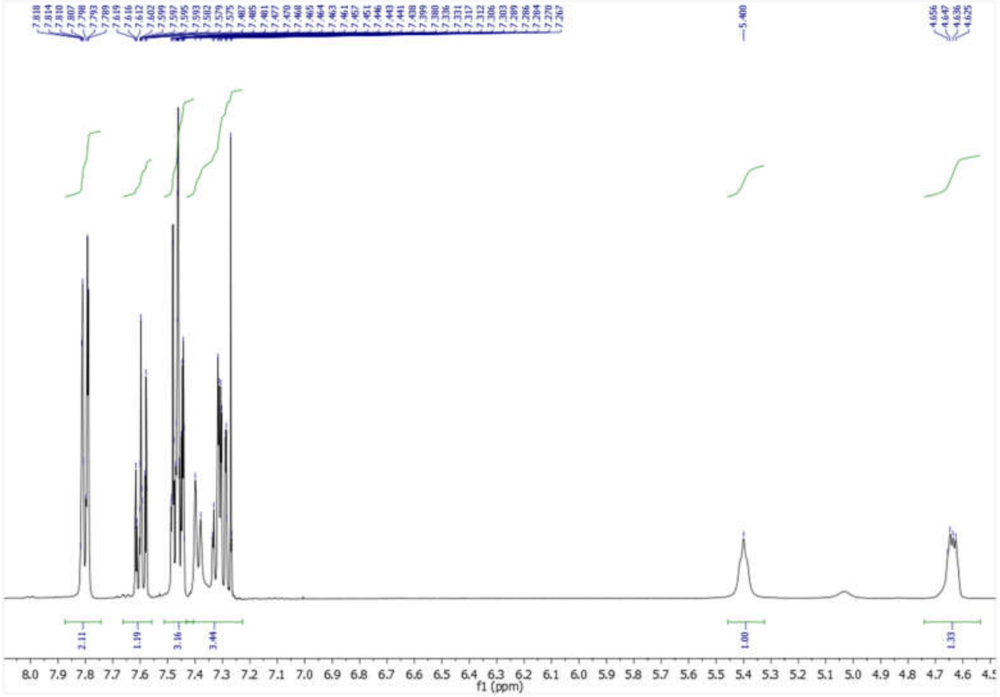

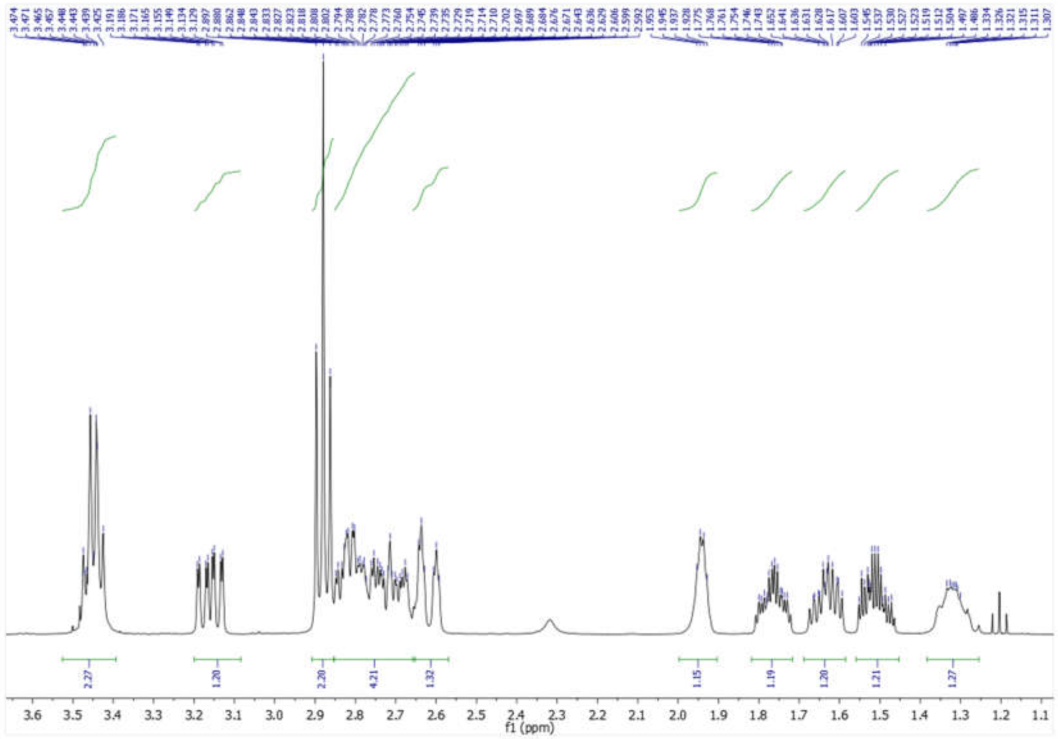

^{13}C (100.16 MHz, $CDCl_3$)

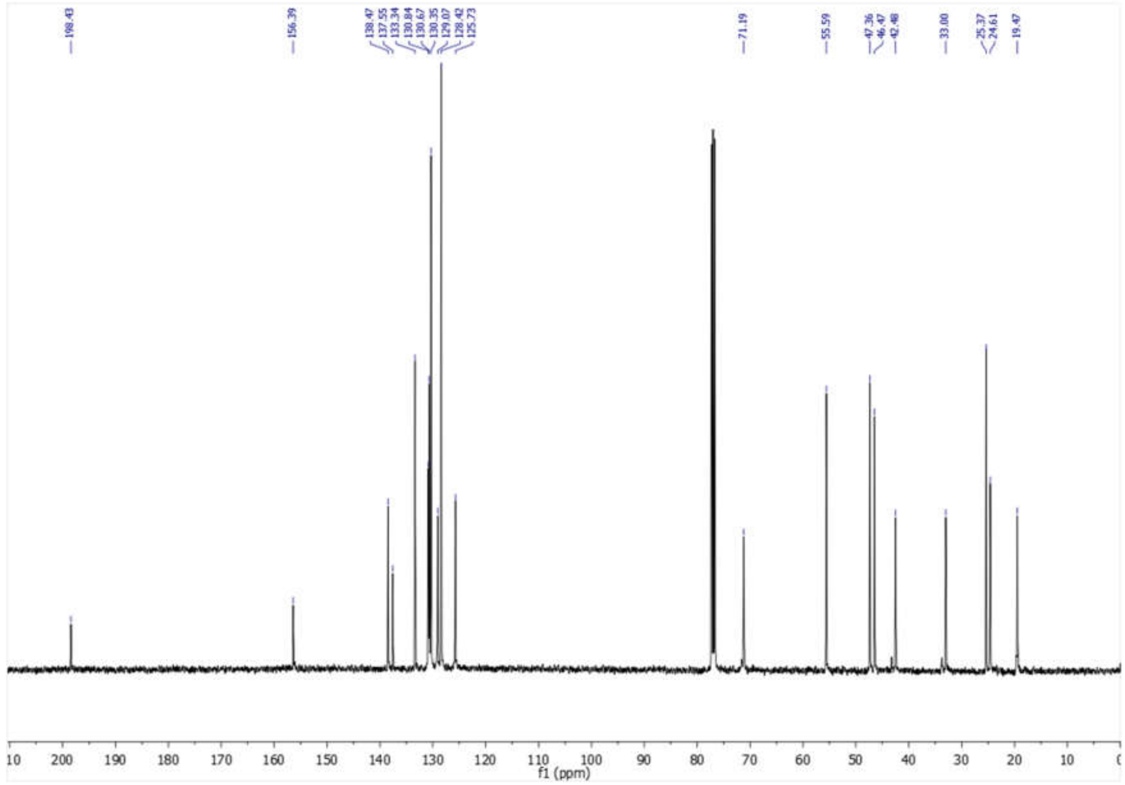

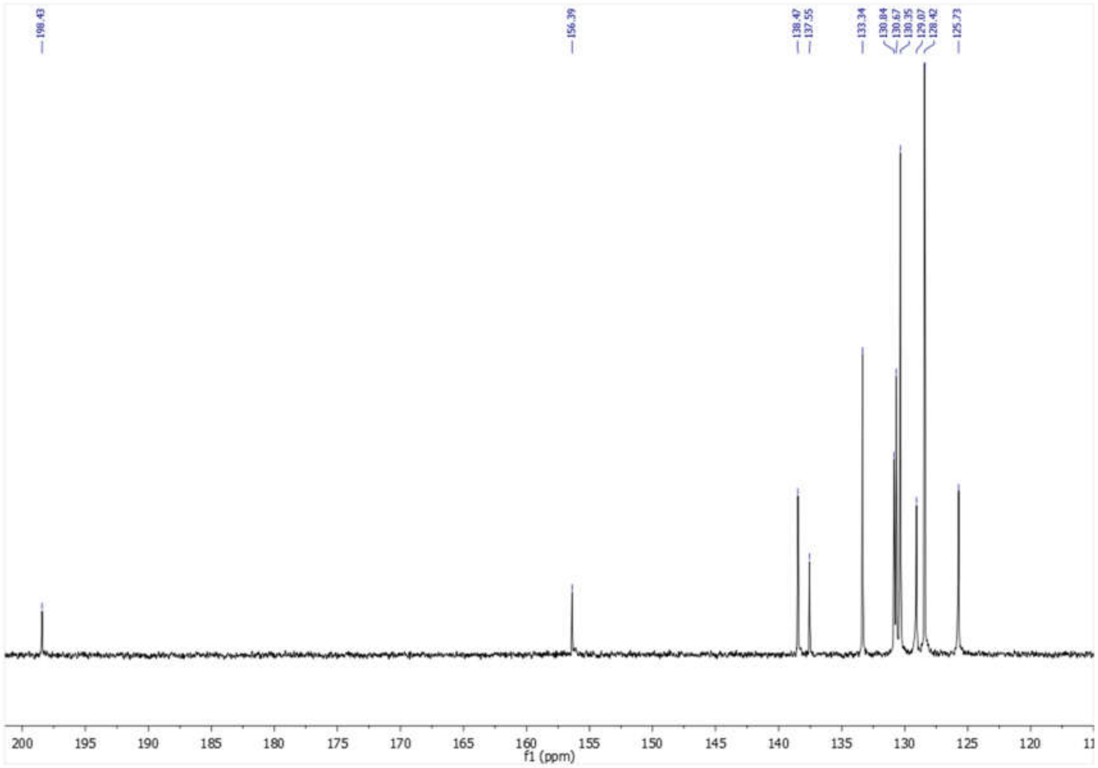

DEPT (400 MHz, $CDCl_3$)

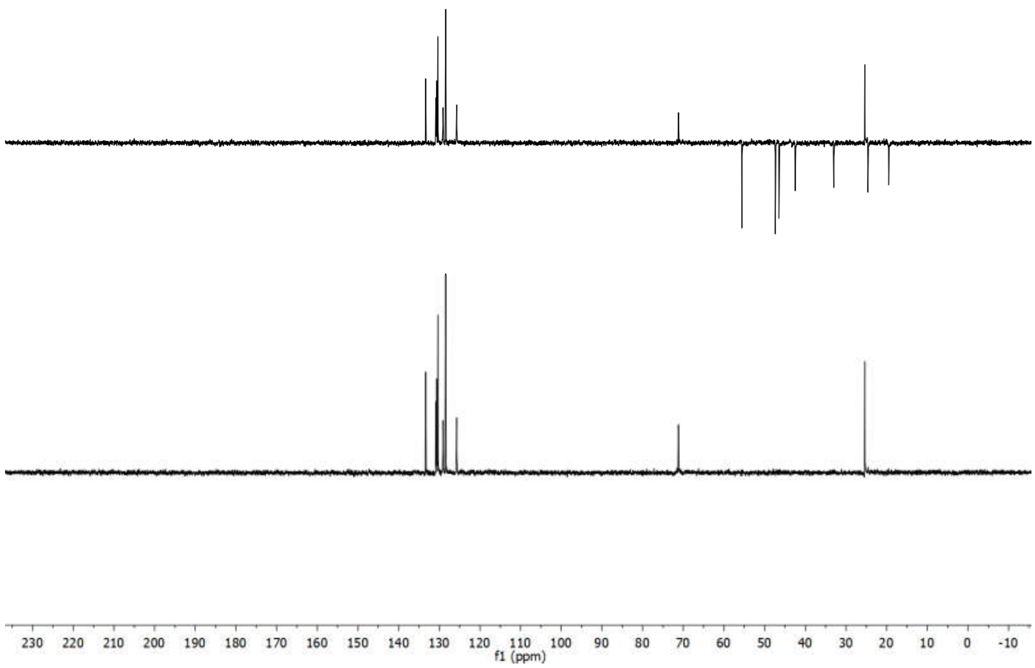

HSQC (400 MHz, $CDCl_3$)

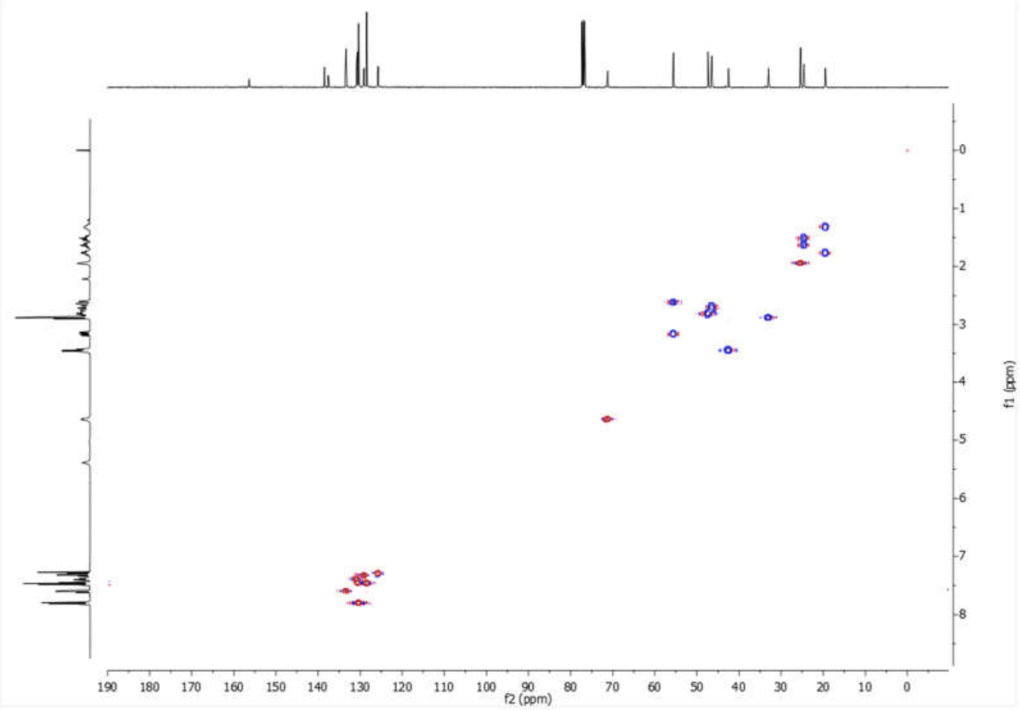

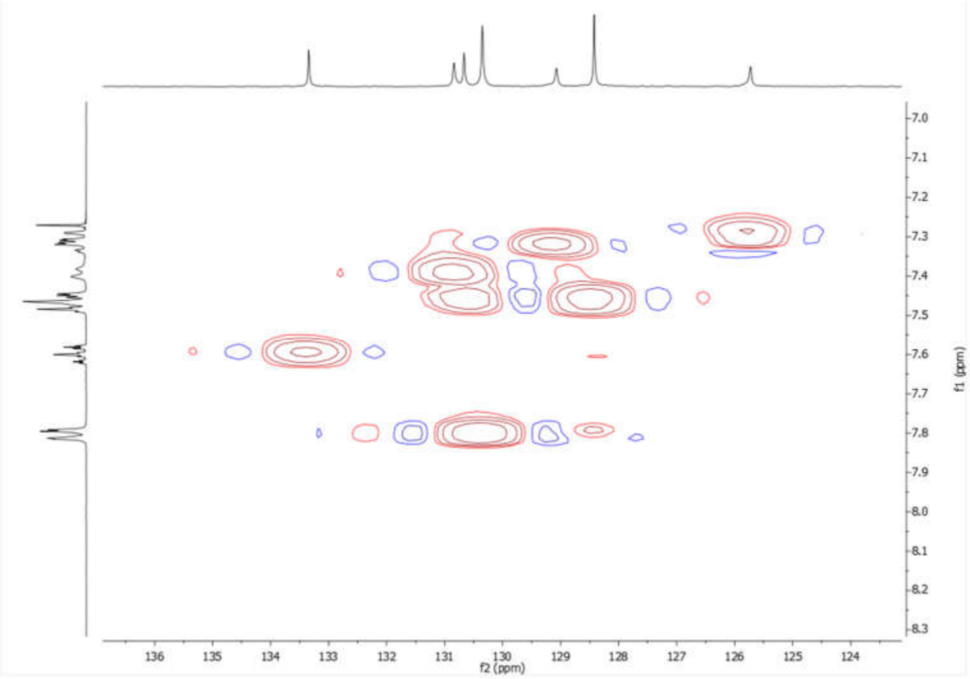

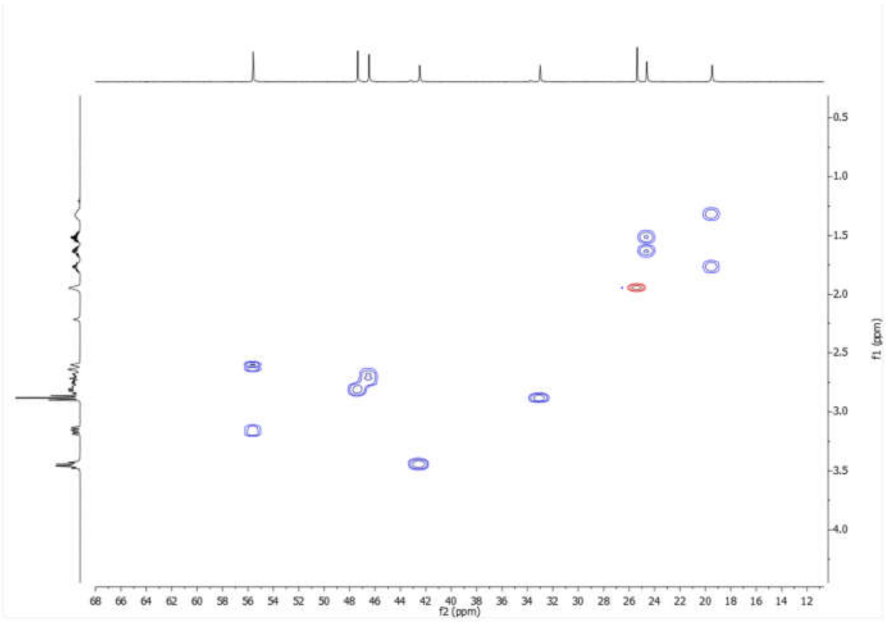

COSY (400 MHz, $CDCl_3$)

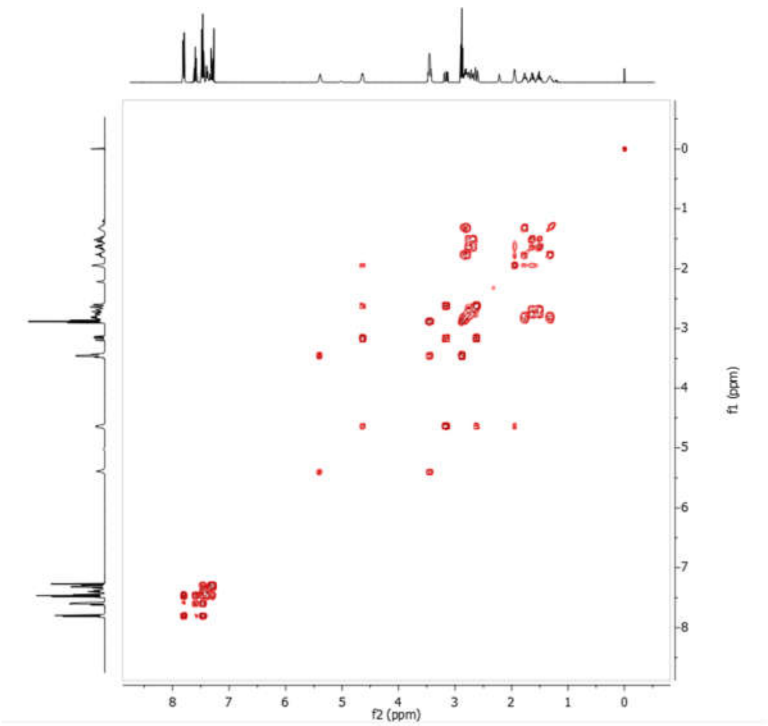

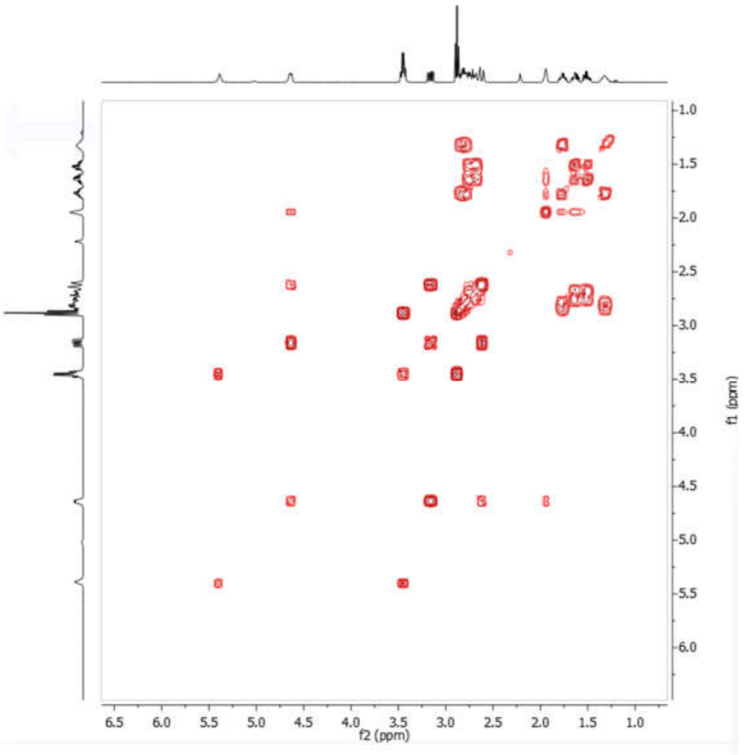
f2 (ppm)
f1 (ppm)

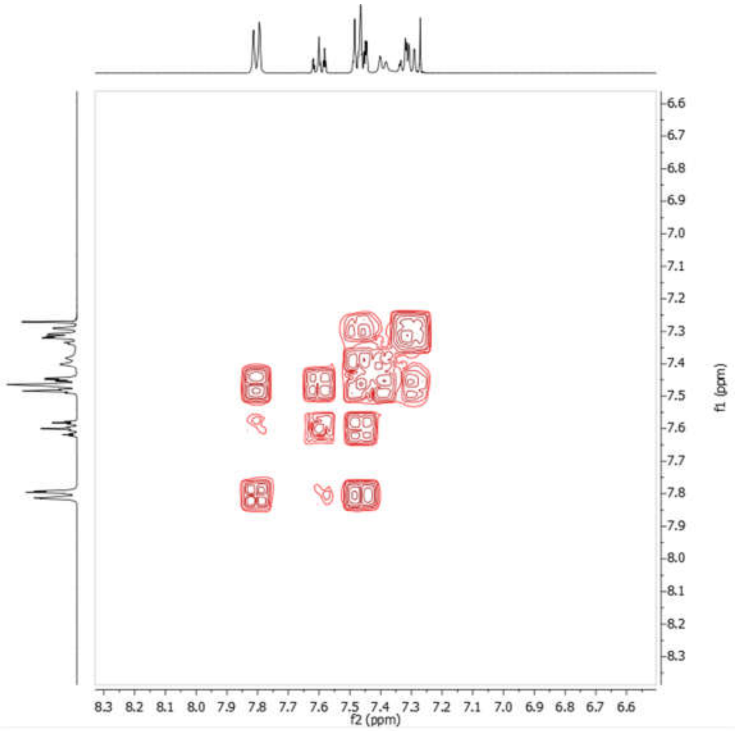
f2 (ppm)
f1 (ppm)

IR (KBr) spectrum of Impurity K

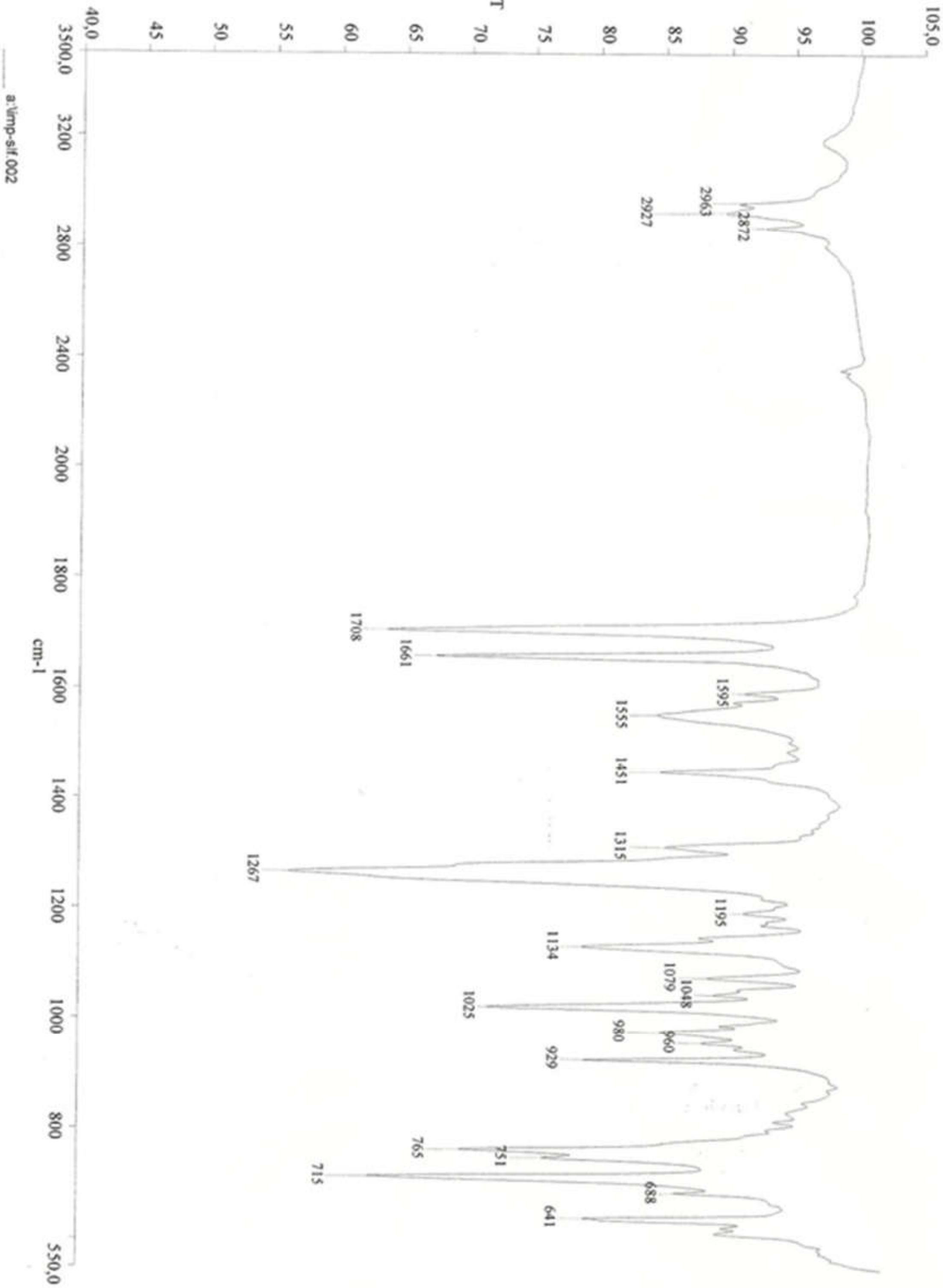

GC/MS (EI) of Impurity K

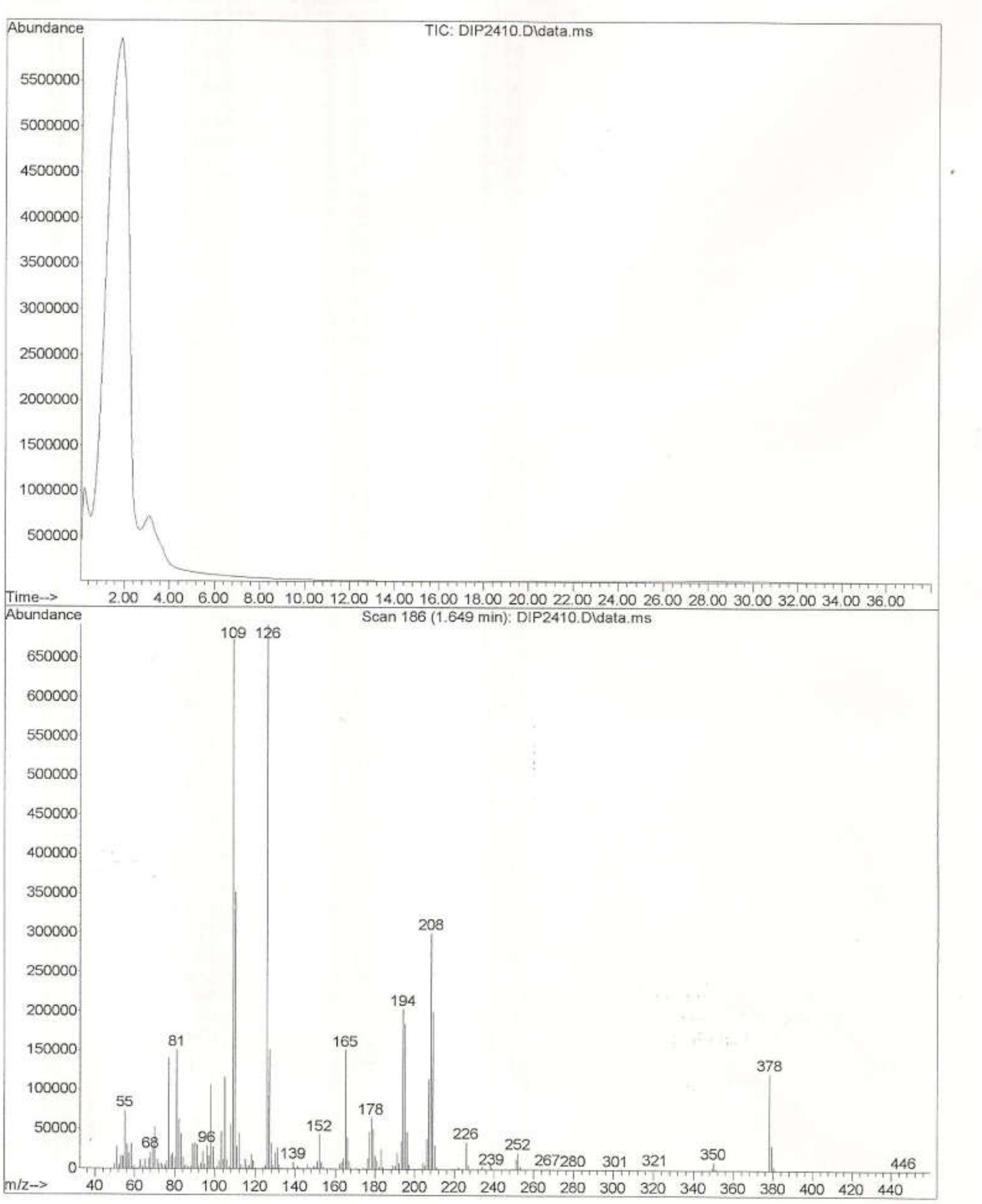

Scan 186 (1.649 min): DIP2410.D\data.ms
IMP-SLF

MASSA	ABUNDANCIA	PERCENTATGE	%Pic Base
*******	************	*************	***********
51.1	27776	0.76	4.02
53.1	15482	0.42	2.24
54.1	16333	0.45	2.36
55.1	72648	1.99	10.50
56.1	29928	0.82	4.33
57.1	19808	0.54	2.86
58.1	31864	0.87	4.61
63.0	11372	0.31	1.64
65.1	11764	0.32	1.70
67.1	13129	0.36	1.90
68.1	20560	0.56	2.97
69.1	29856	0.82	4.32
70.1	52136	1.43	7.54
71.1	11581	0.32	1.67
73.1	7325	0.20	1.06
76.1	9901	0.27	1.43
77.1	139648	3.83	20.19
78.1	16656	0.46	2.41
79.1	19960	0.55	2.89
80.1	13909	0.38	2.01
81.1	150592	4.13	21.77
82.1	61824	1.69	8.94
83.1	43872	1.20	6.34
84.1	14830	0.41	2.14
89.1	30792	0.84	4.45
90.1	32184	0.88	4.65
91.1	29472	0.81	4.26
93.1	7100	0.19	1.03
94.1	21856	0.60	3.16
96.1	28576	0.78	4.13
97.1	16354	0.45	2.36
98.1	106016	2.91	15.33
99.1	27424	0.75	3.97
102.1	10578	0.29	1.53
103.1	46792	1.28	6.77
104.1	11507	0.32	1.66
105.0	115688	3.17	16.73
106.1	11124	0.30	1.61
108.1	55752	1.53	8.06
109.1	672704	18.44	97.26
110.1	351360	9.63	50.80
111.1	28104	0.77	4.06
112.1	44752	1.23	6.47
115.1	12203	0.33	1.76
118.1	18360	0.50	2.65
119.1	10235	0.28	1.48
126.1	691648	18.96	100.00
127.1	151552	4.15	21.91
128.1	32184	0.88	4.65
130.1	20920	0.57	3.02
131.1	26648	0.73	3.85
139.1	8855	0.24	1.28
151.1	9920	0.27	1.43
152.1	43688	1.20	6.32
153.1	8956	0.25	1.29
163.1	9064	0.25	1.31
164.1	12678	0.35	1.83
165.1	151040	4.14	21.84
166.1	39240	1.08	5.67
167.1	9873	0.27	1.43
176.1	13257	0.36	1.92
177.1	46184	1.27	6.68
178.1	64544	1.77	9.33
179.1	50088	1.37	7.24

mifile.txt

180.1	16896	0.46	2.44
181.1	10262	0.28	1.48
183.1	24704	0.68	3.57
191.1	21008	0.58	3.04
192.1	7404	0.20	1.07
193.1	35320	0.97	5.11
194.1	203200	5.57	29.38
195.1	184960	5.07	26.74
196.1	46896	1.29	6.78
204.1	8172	0.22	1.18
206.1	37976	1.04	5.49
207.1	114304	3.13	16.53
208.1	299904	8.22	43.36
209.1	199552	5.47	28.85
210.1	29952	0.82	4.33
226.1	33840	0.93	4.89
238.1	8956	0.25	1.29
251.1	13142	0.36	1.90
252.1	20592	0.56	2.98
350.1	10375	0.28	1.50
378.2	122368	3.35	17.69
379.2	31544	0.86	4.56

HPLC/MS-MS of Solifenacin succinate tablets

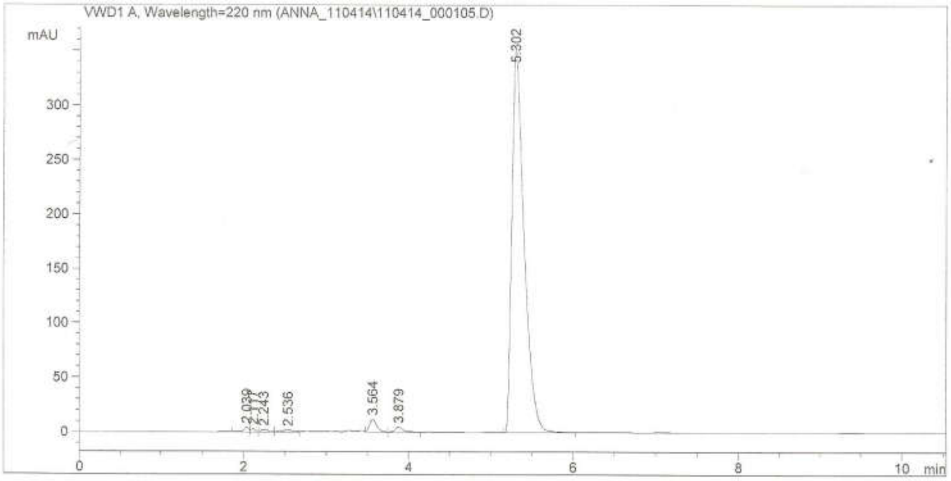


```
=====================================================================
                        Area Percent Report
=====================================================================

Sorted By             :      Signal
Multiplier            :      1.0000
Dilution              :      1.0000
Use Multiplier & Dilution Factor with ISTDs


Signal 1: VWD1 A, Wavelength=220 nm
```

Peak #	RetTime [min]	Type	Width [min]	Area mAU *s	Height [mAU]	Area %
1	2.039	BV	0.0625	18.59203	4.39335	0.4931
2	2.117	VV	0.0551	10.66590	3.07239	0.2829
3	2.243	VV	0.0958	13.93774	2.19820	0.3697
4	2.536	VV	0.1368	20.02751	1.95851	0.5312
5	3.564	VV	0.0929	73.72308	11.86903	1.9553
6	3.879	VV	0.1025	33.33150	4.82188	0.8840
7	5.302	VB	0.1450	3600.16113	353.43842	95.4839
Totals :				3770.43889	381.75177	

```
=====================================================================
                        *** End of Report ***
```

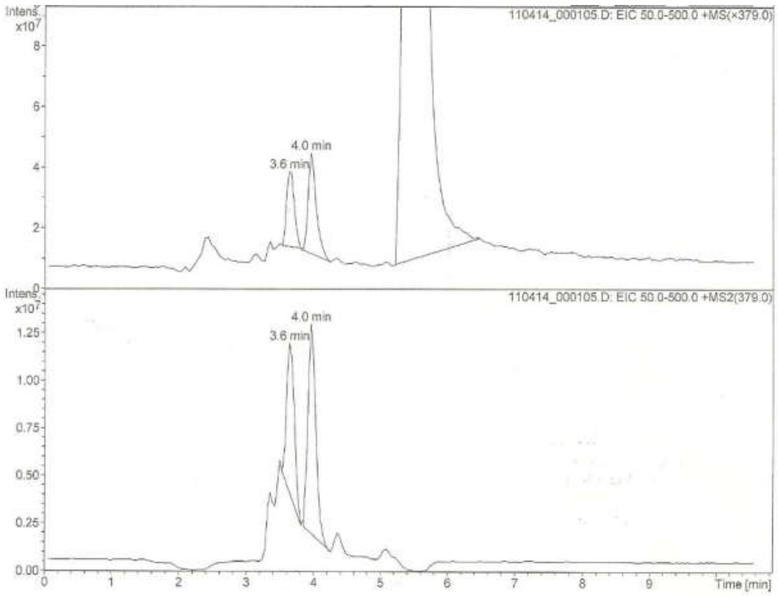

Window Display Report

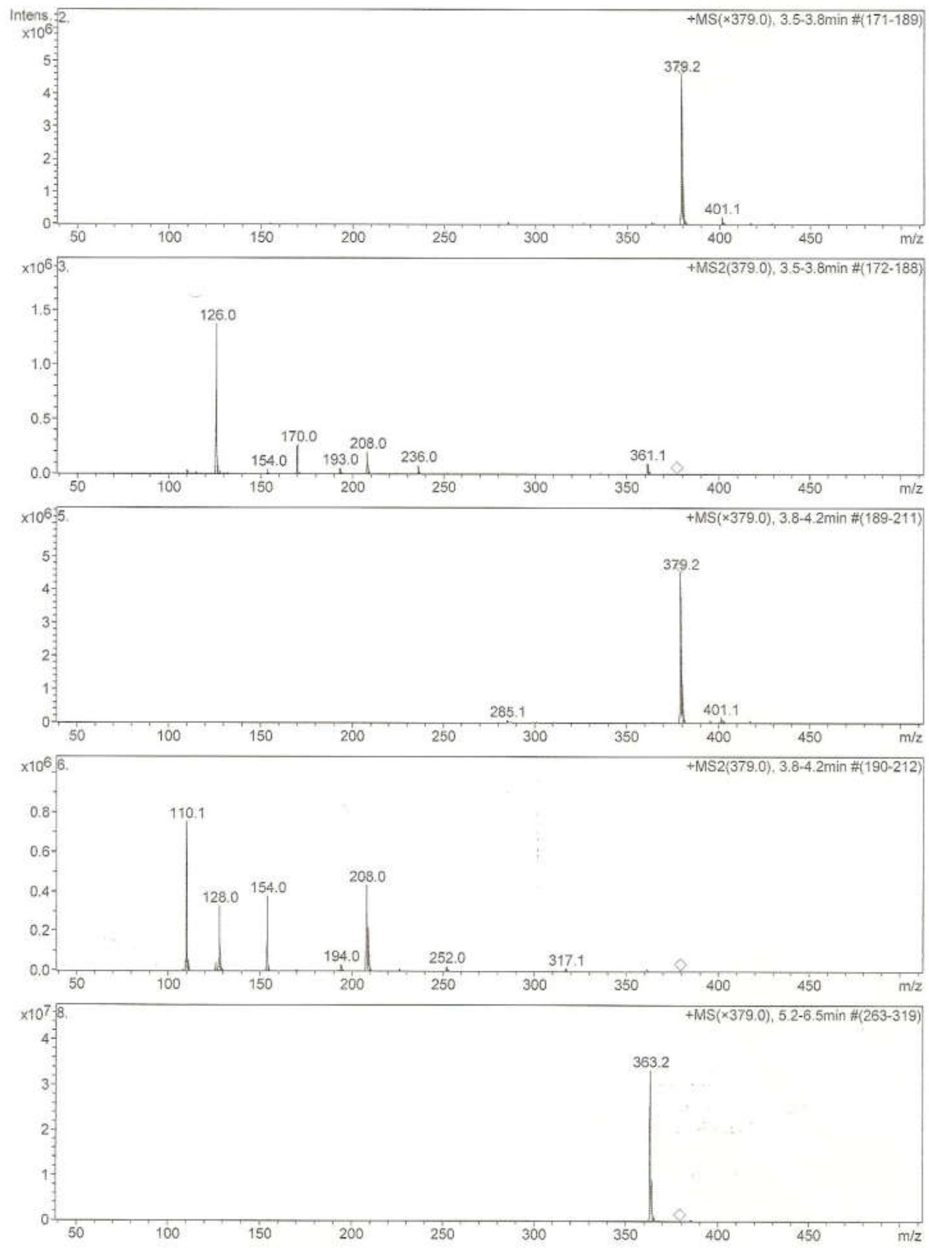

HPLC/MS-MS of Impurity I

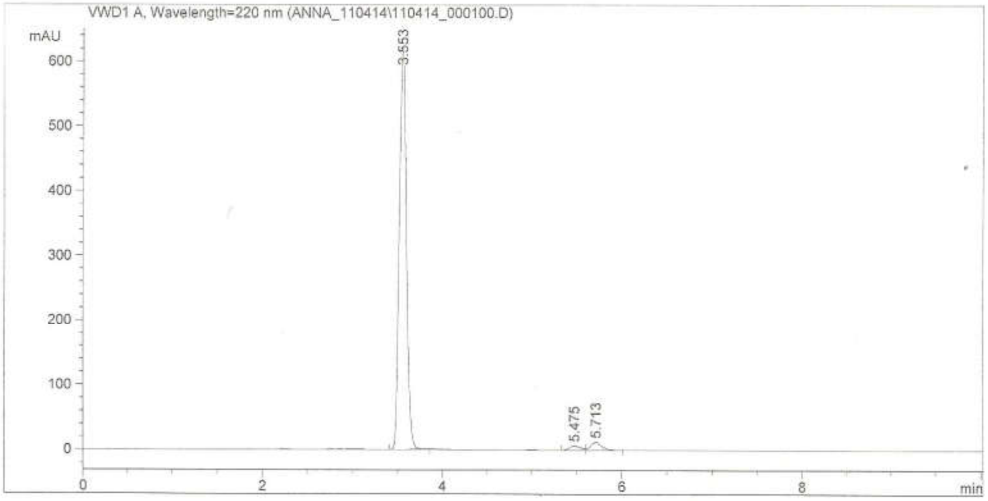


```
=====================================================================
                      Area Percent Report
=====================================================================

Sorted By             :      Signal
Multiplier            :      1.0000
Dilution              :      1.0000
Use Multiplier & Dilution Factor with ISTDs

Signal 1: VWD1 A, Wavelength=220 nm
```

Peak #	RetTime [min]	Type	Width [min]	Area mAU *s	Height [mAU]	Area %
1	3.553	BB	0.0855	3377.74438	619.17914	95.7334
2	5.475	BV	0.1237	51.48433	6.35369	1.4592
3	5.713	VB	0.1255	99.05293	11.99393	2.8074
Totals :				3528.28165	637.52676	

```
=====================================================================
                        *** End of Report ***
```

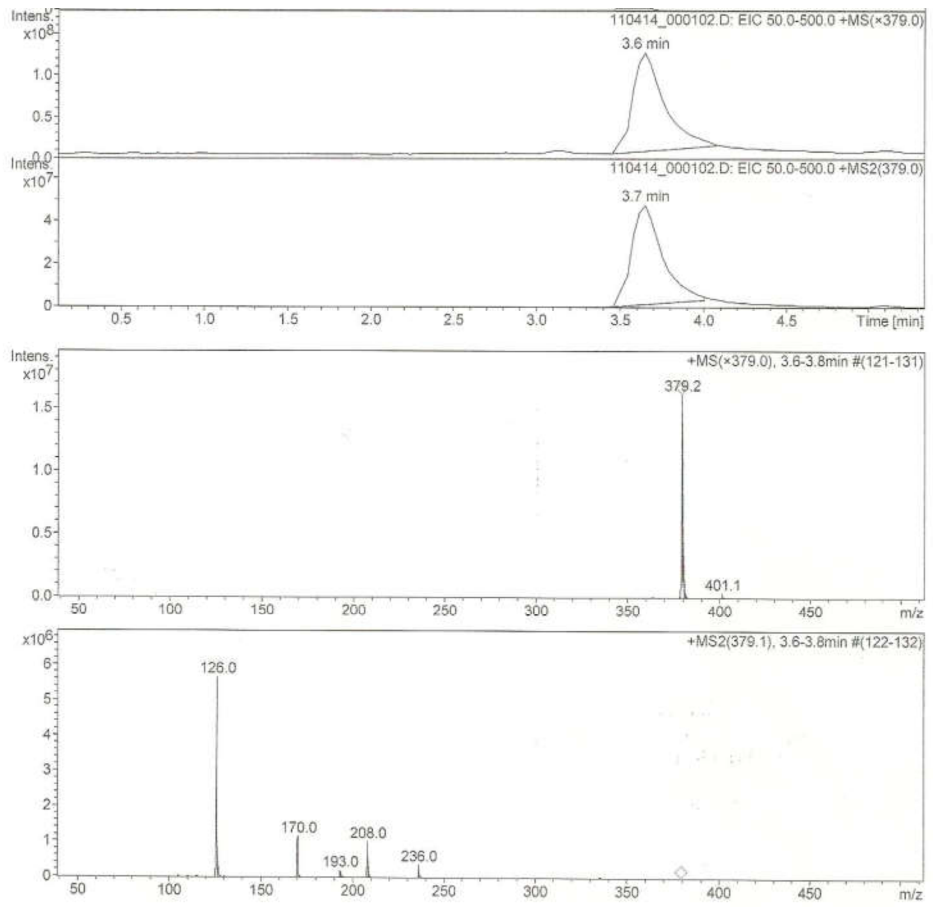
Intens. x10^8
110414_000102.D: EIC 50.0-500.0 +MS(×379.0)
3.6 min
Intens. x10^7
110414_000102.D: EIC 50.0-500.0 +MS2(379.0)
3.7 min
Time [min]
Intens. x10^7
+MS(×379.0), 3.6-3.8min #(121-131)
379.2
401.1
m/z
x10^6
+MS2(379.1), 3.6-3.8min #(122-132)
126.0
170.0
193.0
208.0
236.0
m/z

HPLC/MS-MS of Impurity K

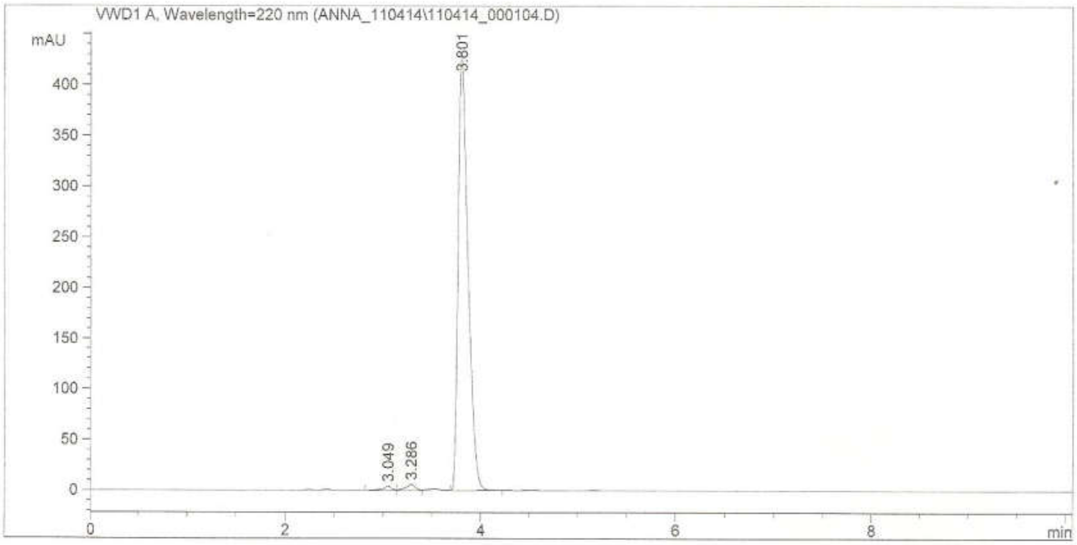


```
=====================================================================
                         Area Percent Report
=====================================================================

Sorted By             :      Signal
Multiplier            :      1.0000
Dilution              :      1.0000
Use Multiplier & Dilution Factor with ISTDs

Signal 1: VWD1 A, Wavelength=220 nm

Peak RetTime Type  Width     Area      Height     Area
  #   [min]        [min]   mAU   *s    [mAU  ]      %
----|-------|----|-------|----------|----------|--------|
   1   3.049 BV    0.0870   23.32405    3.91550   0.7551
   2   3.286 VV    0.0948   38.22941    5.87696   1.2377
   3   3.801 BB    0.1054 3027.20898  430.40933  98.0072

Totals :                  3088.76244  440.20179

=====================================================================
                         *** End of Report ***
```

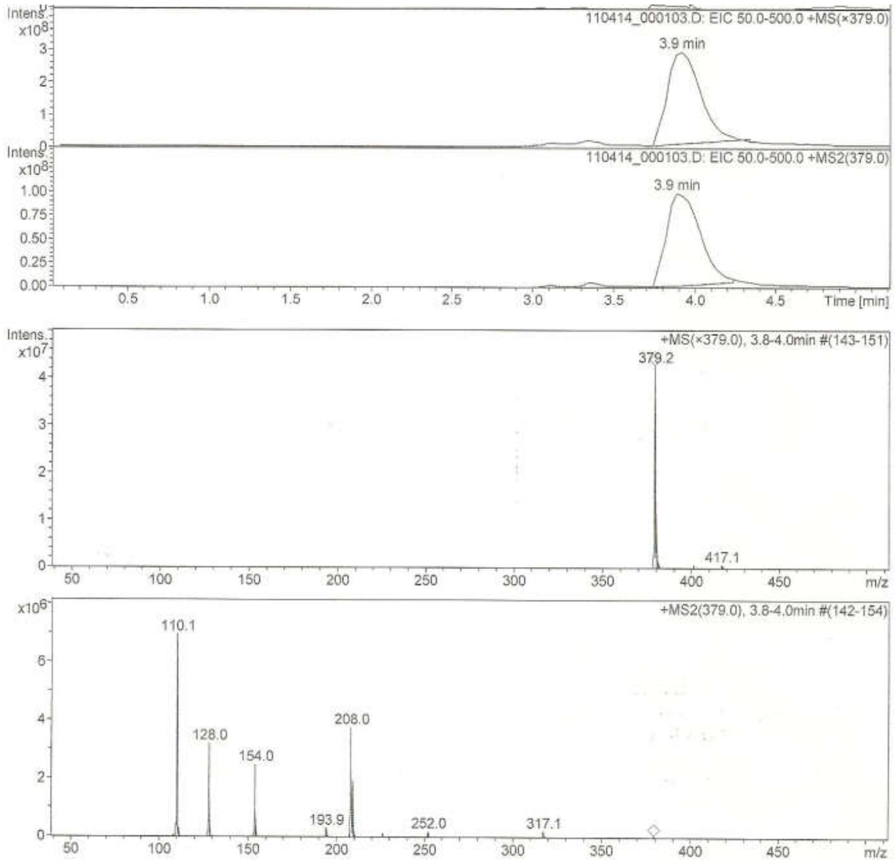
Intens.
x10^8
110414_000103.D: EIC 50.0-500.0 +MS(×379.0)
3.9 min
110414_000103.D: EIC 50.0-500.0 +MS2(379.0)
3.9 min
Time [min]
+MS(×379.0), 3.8-4.0min #(143-151)
379.2
417.1
m/z
+MS2(379.0), 3.8-4.0min #(142-154)
110.1
128.0
154.0
208.0
193.9
252.0
317.1
m/z
